# Sugar-Coated Pills

**Published:** 1877-11-20

**Authors:** 


SUGAR-COATED PILLS.—A contributor in the August number of our journal having claimed priority of manufacture for the sugar-coated pills of Messrs Bullock & Crenshaw, Wm. R. Warner & Co., of Philadelphia, take issue with the writer, and send us the subjoined correspondence, from which it appears that a mistake has been committed, and the honor of priority, etc., rests with the latter firm.

October 4th, 1877.

Mr. W. R. Warner: Dear Sir—I have just read an article in the "Southern Medical Record" by Dr. Wm. A. Green, Macon, Ga., on sugar-coated pills.

Having been your family physician for thirteen years, and during that time known that you have been constantly manufacturing sugar-coated pills, was surprised to see so little credit given you in the article.

I was always under the impression that you were the originator of the process; for at least nineteen years ago I knew of Warner's make of sugar-coated pills.

I understand that the primary board of judges at the Centennial, (consisting of eminent physicians) awarded your house the only medal for sugar-coated pills, and that other firms received a medal from a supplementary board, appointed by the Commission, after the final adjournment of the primary board. Is my information correct?

Respectfully yours,
J. M. BOISNOT, M. D.

*Dear Sir:*—In reply to your favor of 4th inst. it gives us pleasure to state that your impressions as expressed therein are correct.

Wm. R. Warner made sugar-coated pills for Bullock & Crenshaw and supplied that firm with them, from his private Laboratory, for a period of about eight years prior to 1866, at which time no other individual or firm was similarly engaged in Philadelphia or Pennsylvsnia. Wm. R. Warner & Co., were awarded the premium by the primary judges of the Centennial. B. & C. obtained a premium from supplementary judges, appointed by the Commission near the close of the exhibition.

We presume the Doctor used statements furnished him as a basis for his essay, and as to their inaccuracy we have positive proof to exhibit when necessary.

Yours respectfully,
WM. R. WARNER & Co.

*Judges Report.*—"The sugar-coated pills of William R. Warner & Co. are soluble, reliable and unsurpassed in the perfection of sugar-coating, thorough composition and accurate subdivision.

"The pills of PHOSPHORUS, are worthy of special notice. The element is thoroughty diffused and subdivided, yet perfectly protected from oxidation.

A. T. GOSHORN, Director General.
[Attest.] F. R. HAWLEY, President.
F. L. CAMPBELL, [SEAL.]

